# Efficient golden gate assembly of DNA constructs for single molecule force spectroscopy and imaging

**DOI:** 10.1093/nar/gkac300

**Published:** 2022-04-30

**Authors:** Nicholas A W Bell, Justin E Molloy

**Affiliations:** The Francis Crick Institute, London NW1 1AT, UK; The Francis Crick Institute, London NW1 1AT, UK

## Abstract

Single-molecule techniques such as optical tweezers and fluorescence imaging are powerful tools for probing the biophysics of DNA and DNA-protein interactions. The application of these methods requires efficient approaches for creating designed DNA structures with labels for binding to a surface or microscopic beads. In this paper, we develop a simple and fast technique for making a diverse range of such DNA constructs by combining PCR amplicons and synthetic oligonucleotides using golden gate assembly rules. We demonstrate high yield fabrication of torsionally-constrained duplex DNA up to 10 kbp in length and a variety of DNA hairpin structures. We also show how tethering to a cross-linked antibody substrate significantly enhances measurement lifetime under high force. This rapid and adaptable fabrication method streamlines the assembly of DNA constructs for single molecule biophysics.

## INTRODUCTION

Single-molecule methods have enabled deep insights into the biophysics of DNA and DNA–protein interactions. Force spectroscopy techniques such as optical and magnetic tweezers enable the investigation of the effects of tension and torsion in DNA processes (1–3). Control of these parameters has helped unravel key steps in transcription (4,5), helicase-based unwinding (6–8) and chromatin organization (9,10). Alongside force spectroscopy methods, a large variety of techniques have been developed for fluorescence imaging on long DNA molecules in order to track the topology of DNA and the movement of DNA binding proteins. For instance, so-called DNA curtains (11,12) where DNA molecules are stretched across a cover slip surface, permit single molecule visualization of proteins diffusing along the DNA contour and binding to specific locations. Force spectroscopy and fluorescence imaging can also be combined, for example using dual beam optical traps and confocal or epifluorescence imaging (13,14).

The generation of defined DNA substrates is an important pre-requisite for all single molecule techniques using either force spectroscopy or imaging. For many applications these substrates must be on the order of kilobases in length in order to create space between the coupling surfaces or to enable fluorescence tracking of isolated molecules. λ-DNA is frequently used since it provides an inexpensive source of long (48.5 kb) and pure double-stranded DNA with 12 base overhangs for straightforward attachment of labelled oligonucleotides. Nicking endonuclease based methods can be used to introduce modification at specific sites on λ-DNA (15,16). However, it is not straightforward to insert specific sequences or structures at arbitrary locations since nicking endonucleases are limited to certain sites along the DNA. For the generation of shorter constructs with a wide variety of structural features and labels, a range of methods based on PCR digestion-ligation and single-stranded annealing have been described (17–23). However, these methods typically involve multiple rounds of digestion, purification and ligation which is time consuming and can limit the synthesis yield. Annealing of multiple synthetic oligonucleotides to a single-stranded phage genome enables positioning of labels along the DNA contour but does not produce a ligated product which is required in many single molecule techniques (24). Overall, there remains an unmet need for a simple and adaptable method for creating double-stranded DNA constructs which are kilobases in length and which can contain synthesized oligonucleotides and DNA breaks at specific designed locations.

In this paper, we develop a high-yield fabrication method based on golden gate assembly for the construction of long DNA constructs, which combine PCR amplicons and synthetic oligonucleotides. We leverage recent profiling of ligation accuracy by T4 DNA ligase (25) to efficiently construct torsionally constrained DNA up to 10 kb in length as well as DNA hairpin structures which contain multiple synthetic oligonucleotides at designed positions. We also show that chemical cross-linking of surface immobilized antibodies can significantly enhance the lifetime of DNA tethers labelled with hapten-modified nucleotides. The assembly strategies that we describe greatly simplify the construction of diverse DNA templates required for single molecule studies.

## MATERIALS AND METHODS

### PCR synthesis

Biotin and digoxigenin labelled DNA (so-called ‘DNA handles’) were synthesized by incorporation of modified dUTPs using Taq polymerase (TaqPCR kit, NEB). The PCR mixture was setup according to the manufacturer's instructions. For synthesizing biotin labelled DNA, biotin-11-dUTP (Jena Bioscience) was added to the PCR mixture at a ratio of 1:8 of biotin-dUTP:dTTP. For synthesizing digoxigenin labelled DNA, dig-11-dUTP (Jena Bioscience) was added to the PCR mixture at a ratio of 1:8 of dig-dUTP:dTTP. The PCR for handle synthesis amplified an 860 bp section of λ-DNA (NEB). Unlabelled PCR sections were synthesized using a Taq based master mix (LongAmp Hot Start Master Mix, NEB). After the PCR amplification, samples were purified by a spin column (Qiaquick, Qiagen) according to the manufacturer's instructions. The primers used for each structure described in this study are given in the SI (Supplementary Tables S1–S8). The PCR protocol was optimized so that a single strong band was observed by gel analysis (see Supplementary Figure S1) and therefore gel purification was not needed before the golden gate assembly step.

### Assembly of oligonucleotide parts

All oligonucleotides were purchased from Integrated DNA Technologies (IDT). Each oligonucleotide part was separately assembled by mixing the component oligos to a final concentration of 10 μM in IDT duplex buffer (30 mM HEPES, pH 7.5; 100 mM potassium acetate) and annealing from 70°C to room temperature over 30 min. The specific sequences of each oligonucleotide part are given in the SI (Supplementary Tables S1–S8).

### Golden gate assembly

DNA construct designs were assembled by mixing PCR parts and oligonucleotide parts together with BsaI (BsaI-HFv2, NEB) and T4 DNA ligase (NEB) in 1× T4 DNA ligase buffer (NEB). The assembly was carried out in a single tube of 20–40 μl volume with final concentrations of 1 U/μl BsaI-HFv2 and 20 U/μl T4 DNA ligase. The total DNA concentration in the golden gate reaction was 30–100 ng/μl and was kept as high as possible to maximize the ligation efficiency. The parts were mixed such that the stoichiometry was an increasing ratio from the central part in the design to the outside parts (see SI for full details). The mixture was incubated for 3 h cycling between 37°C and 16°C every 5 min.

### Agarose gel analysis

Assembly products were analysed on a 1% agarose gel with 1xTAE and run at 80 V. For imaging gels, the DNA was post-stained with GelRed (Biotium) and visualized with a UV illuminator. Three hours run time was sufficient for separating the main band at 10 kb from the side product at 9 kb (Supplementary Figure S2). Extension of our method to longer DNA lengths would benefit from using longer PCR parts to enable straightforward separation of the side products. For extraction, the gel was stained with GelGreen (Biotium) and the bands cut out under blue light to prevent UV induced damage of the DNA. The DNA was then purified with a commercial silica bead kit (Qiaex II, Qiagen). The DNA was quantified with a Qubit fluorometer (ThermoFisher) with typical yields of 0.5–2 ng/μl.

### Flow cell fabrication

Microfludic flow cells with gravity controlled perfusion rates were constructed as previously described (26). Briefly, a glass cover slip was coated on one surface with nitrocellulose using a spin coater. A flow cell was then formed by sandwiching a channel formed in double-sided adhesive tape between this nitrocellulose coated cover slip and a second uncoated cover slip. The channel formed had a volume of 5 μl.

### Magnetic tweezers measurements

3 μm latex spheres with sulfate surface groups (LB30, Sigma Aldrich) were initially introduced into the flow cell in a buffer of 1 mM Tris pH 7.5 and allowed to bind non-specifically to the nitrocellulose surface. These latex spheres act as fiducials for drift correction during an experiment. Antibody solution at 0.7 mg/ml in PBS was then added and incubated for 5 minutes before washing with ∼20 channel volumes of PBS (the channel volume was 5 μl). For Figure 3A, we used fab fragments of sheep polyclonal anti-digoxigenin (11214667001, Sigma Aldrich). For all other figures, we used full-length IgG anti-digoxigenin antibodies (11333089001, Sigma Aldrich). For the glutaraldehyde treatment shown in Figure 3C, glutaraldehyde (G7776, Sigma Aldrich) at 2% in PBS was freshly prepared from a stock solution of 70% and added to the flow cell for 5 min followed by flowing through ∼10 channel volumes of 1 M Tris–HCl pH 7.5 to quench the cross-linking reaction. The flow cell surface was subsequently blocked by 20 mM Tris–HCl pH 7.5, 150 mM NaCl, 10 mg/ml BSA (A0281, Sigma-Aldrich), 10 mg/ml β-casein (C6905, Sigma-Aldrich) which was incubated for 5 min. The gel purified DNA constructs were mixed with either 2.8 μm diameter (M-280 streptavidin Dynabeads, ThermoFisher) or 1 μm diameter (MyOne streptavidin T1 Dynabeads, ThermoFisher) magnetic beads. The 1 μm diameter beads were used for Figure 2G and the 2.8 μm beads were used for Figures 2D, E, 3D, 4D, 5E, F. The ratio of DNA:beads was approximately 0.05 ng DNA for 5 μl M280 Dynabeads stock. The mixture was incubated for 15 minutes before pull down of the beads, washing with measurement buffer and introducing into the flow cell. All measurements were performed in a buffer of 20 mM Tris–HCl pH 7.5, 150 mM NaCl, 1 mg/ml BSA, 1 mg/ml β-casein except for the PcrA unwinding experiments which used 1× T4 DNA ligase buffer (NEB). PcrA was added at a concentration of 0.2 nM. Purified PcrA was a kind gift from the Martin Webb lab. The imaging setup used bright field illumination on an inverted Nikon Eclipse TE2000 microscope with a 60× Nikon oil immersion objective. The tracking algorithm and microscope setup has been previously described (27). We observed minimal non-specific binding of magnetic beads to the flow cell surface (Supplementary Figure S3).

### Flow-stretching TIRF microscopy

For combined flow stretching and TIRF imaging, single molecule tethers were prepared as described above with 1 μm magnetic beads (MyOne streptavidin T1 dynabeads, ThermoFisher) and the DNA was stretched perpendicular to the optical axis using a flow rate of ∼0.5 μl/s. The imaging buffer contained 20 mM Tris–HCl pH 7.5, 150 mM NaCl, 1 mg/ml BSA, 1 mg/ml β-casein, 20 nM sytox orange (ThermoFisher). The sample was illuminated with a 532 nm laser and imaged with an Andor Ixon EMCCD (exposure time was 50 ms).

## RESULTS

### Design principles for assembly

**Figure 1.**
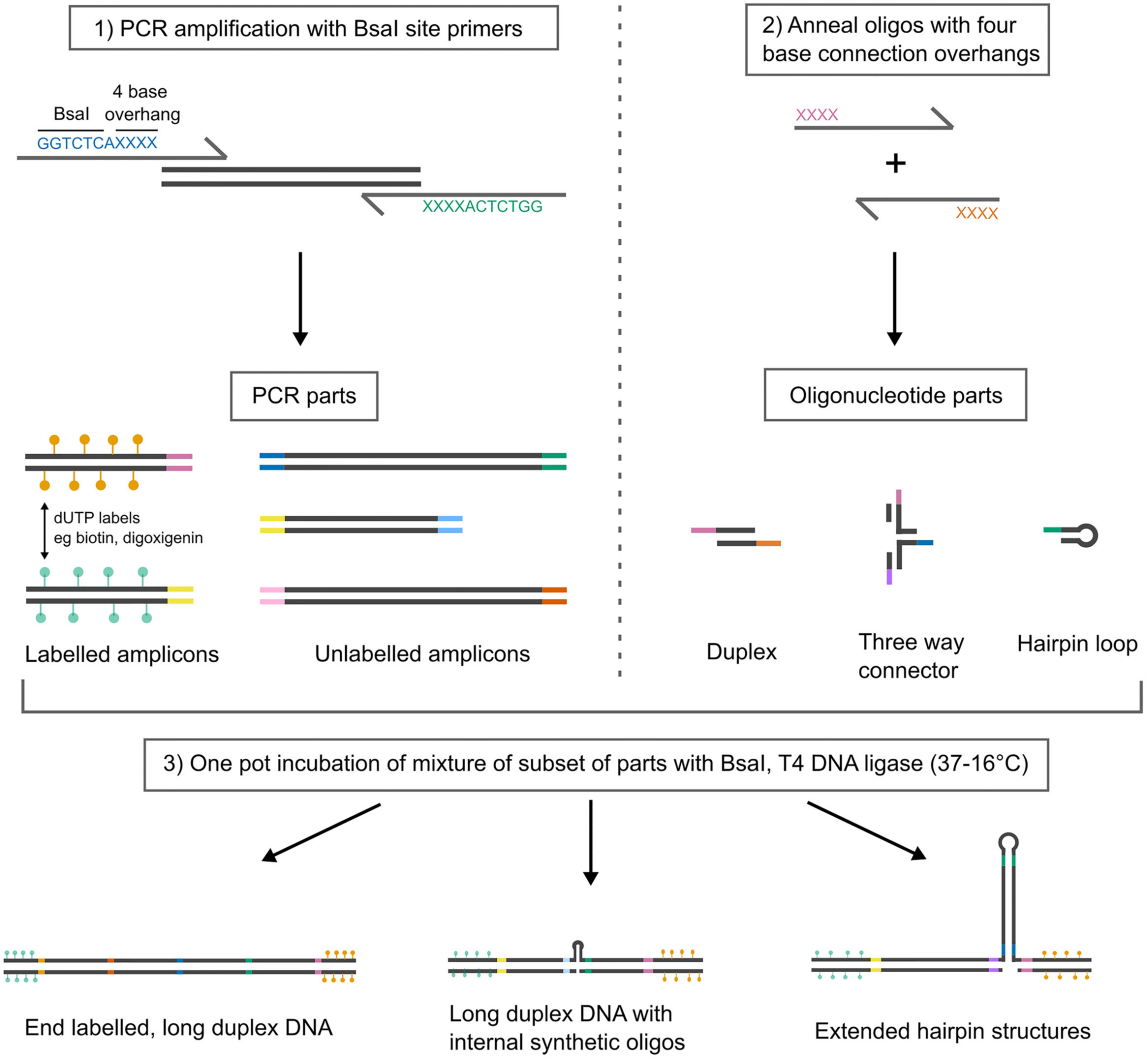
Workflow for generating long duplex DNA and hairpin structures via golden gate assembly. PCR amplicons are generated with primer overhangs that code for the BsaI recognition site and a unique four letter sequence generated as a 5′ overhang after BsaI digestion (each four base overhang containing sequence and its reverse complement is represented by a different colour). For attachment to surfaces or beads, labelled dUTPs are included in the amplification step. Separately, synthetic oligonucleotide parts are annealed together to form different structures such as duplexes, connectors and hairpin loops. These oligonucleotide parts have four base overhangs designed to base pair with specific PCR amplicons. To form the final construct design, specific subsets of the PCR parts and oligonucleotide parts are incubated together with BsaI and T4 DNA ligase in a one pot reaction.

In Figure 1, we show the general workflow we designed for assembling DNA constructs from both PCR amplicons and synthetic oligonucleotides using golden gate assembly. Golden gate assembly relies on the use of Type IIs restriction enzymes which cut upstream from their recognition sites. For instance, the widely used Type IIs enzyme BsaI leaves a four base overhang which is one base upstream from its seven base recognition site. In the first step of our method, long double-stranded (ds) DNA sections are generated by PCR using primers with recognition sites for the BsaI together with a four base overhang sequence for ligation to neighbouring PCR parts or oligonucleotide parts in the design. These PCR parts were generated using a template of λ-DNA. The four base overhang produced by BsaI gives a potential of 256 different sequences which can be used for ligation. Certain combinations of these sequences are known to cause off target ligation by T4 DNA ligase. We used recent developments in high accuracy profiling of T4 DNA ligase fidelity in order to choose a set of four base overhangs from BsaI digestion, which minimizes the frequency of off target ligation (25,28) (see SI for full sequence details). After PCR amplification, the amplicons are purified with a standard spin column. The oligonucleotide parts are separately assembled by annealing the components and contain four base overhangs for attachment to the PCR made sections. To form the final desired structure, specific subsets of these PCR parts and oligonucleotide parts are mixed together with BsaI and T4 DNA ligase in a single tube. The concerted actions of BsaI and T4 DNA ligase digests the PCR amplicons at their specific recognition sites and ligates the neighbouring fragments and oligonucleotide part design. As per the golden gate assembly idea (29), the position of the BsaI recognition sites on the outside of the primers means that the reaction proceeds directionally towards the final designed product. Although other techniques for directional assembly exist, for instance using incomplete restriction sites (30–32), the golden gate technique has the ability to join many different parts. For example, a recent study showed the construction of vectors from up to 52 separate DNA sections (33).

### 10 kb design for torsionally-constrained DNA

**Figure 2.**
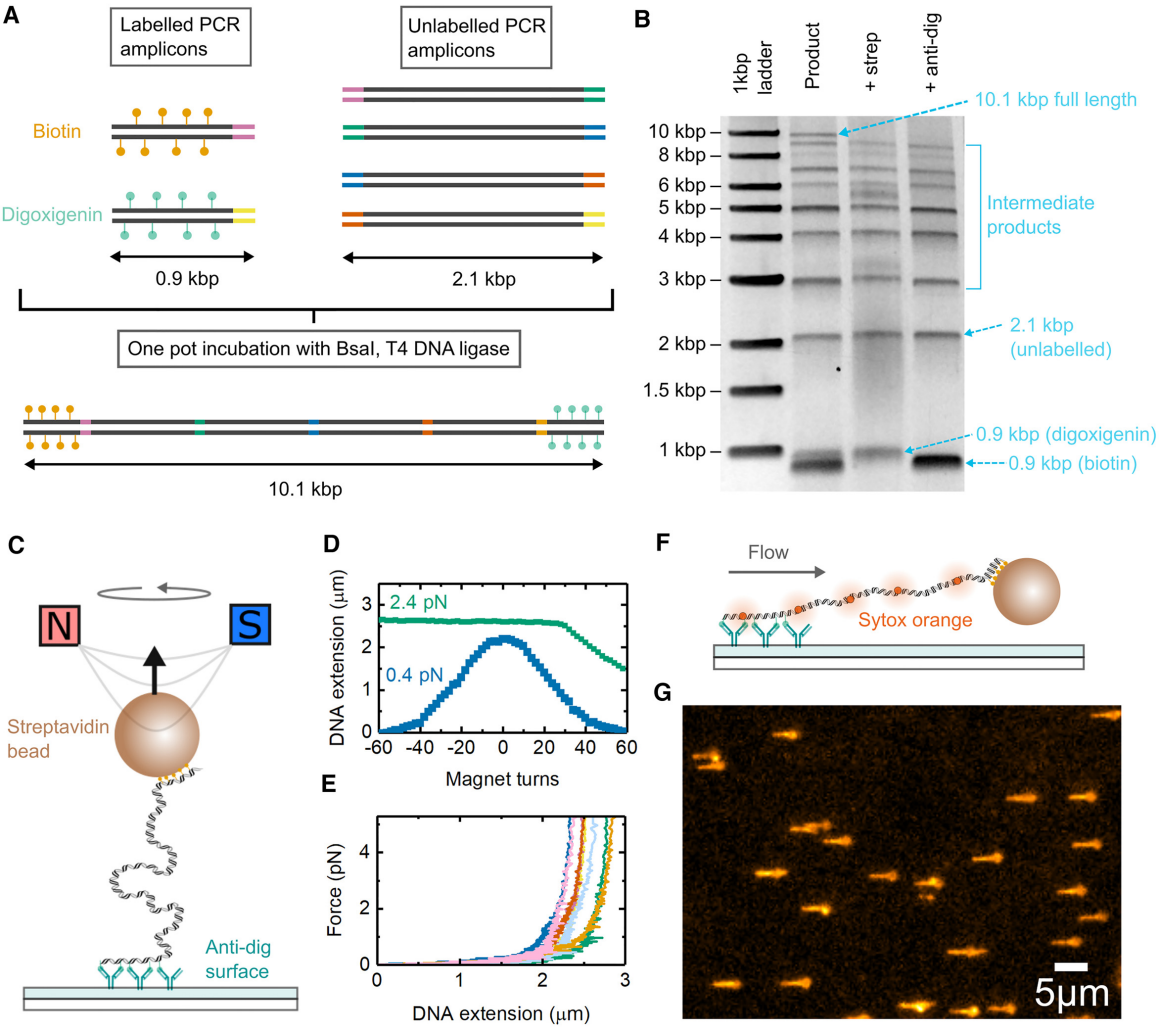
Fabrication of torsionally-constrained DNA with length up to 10 kb by golden gate assembly. (**A**) 0.9 kbp DNA handles were made by PCR using either biotin or digoxigenin dUTP incorporation. Unlabelled PCR sections of 2.1 kb with encoded BsaI sites were separately made. The designed structure after joining of the purification PCR parts by golden gate assembly is 10.1 kb in length. (**B**) Agarose gel image of the product from the golden gate assembly reaction. The band at ∼10 kb reacts with both streptavidin and anti-digoxigenin indicating successful dual labelling. (**C**) Schematic of magnetic tweezers used for force spectroscopy measurements. (**D**) Example torsional response of single DNA tether showing characteristic supercoiling at low force. (**E**) Force extension curves of six DNA tethers in different colours. (**F**) Schematic of flow stretching of DNA molecules. (**G**) TIRF image of multiple beads stretched by a flow of 0.5 μl/s.

Initially we tested the achievable yield for a long duplex DNA construct constructed from multiple parts using golden gate assembly. We designed a DNA construct which was 10.1 kb in total length, combining four 2.1 kb PCR amplicons and two 0.9 kb PCR amplicons which were labelled with either biotin or digoxigenin nucleotides incorporated during the amplification. These six parts were designed with five connection points represented by the different colours in Figure 2A. The six parts were mixed together with BsaI and T4 DNA ligase and incubated for 3 h cycling between 37°C and 16°C every 5 min. The product was analyzed by agarose gel electrophoresis (Figure 2B). A band at ∼10 kb was observed together with bands corresponding to the labelled PCR amplicons, unlabelled 2.1 kb sections and a number of intermediate products (a full assignment of all bands is given in Supplementary Figure S2). The band at ∼10 kb disappeared after addition of either streptavidin or anti-digoxigenin confirming that it is labelled with both biotin and digoxigenin. From image analysis of the gel, the amount of DNA in the final product band is 3% of the total DNA added to the gel lane and the concentration of 10.1 kbp product after golden gate assembly was 3 ng/μl (the total reaction volume was 25 μl of which 1 μl was used for gel analysis). We found that incubation beyond 3 h did not significantly increase the proportion of final product (Supplementary Figure S1). The final yield is likely limited by the presence of off-target amplification products during the PCR step. However, this concentration of final product is more than sufficient for most single molecule experiments – for instance for each magnetic tweezers experiment we use ∼0.05 ng of DNA during the incubation step with magnetic beads prior to adding to the flow cell.

The band at ∼10 kb was gel purified and tested using magnetic tweezers. A microfluidic flow cell was prepared with a surface coated with nitrocellulose and anti-digoxigenin antibodies. Streptavidin coated magnetic beads were incubated with the purified DNA and then added to the flow cell to form a DNA tether between the bead and the coverslip surface (Figure 2C). Figure 2D shows an example of a torsionally-constrained tether which exhibits DNA supercoiling upon magnet rotation (34). We prepared three independent batches of the DNA and found that the percentage of torsionally-constrained DNA molecules was 40 ± 12% (mean ± s.d., *n* = 180 total tethers tested) comparable to previous literature reports using a variety of methods (18,30). Figure 2E shows several examples of force–extension curves of DNA tethers consistent with the 8.4 kb (2.9 μm contour length) of DNA between the two labelled handles. The variability in offset of the force–extension curves is a known phenomenon in magnetic tweezers due to the off centre attachment of DNA resulting from alignment of the magnetic particles’ easy axis with the magnetic field lines (35–37). We also visualized the DNA tethers by TIRF microscopy and a simultaneous fluid flow to stretch the DNA perpendicular to the optical axis (Figure 2F). This showed a highly monodisperse collection of DNA constructs (Figure 2G).

### Enhanced DNA tether strength with antibody cross-linking

Heterofunctional labelling of DNA by incorporation of digoxigenin and biotin modified nucleotides is a widely used method for tethering DNA in magnetic and optical tweezers studies. Biotin binds streptavidin rapidly (*k*_on_ ∼ 10^7^ M^–1^ s^–1^) (38) and forms a very stable bond with a rupture force on the order of hundreds of pN (39). Digoxigenin–anti-digoxigenin interactions also show fast binding kinetics with on-rates of ∼10^7^ M^–1^ s^–1^—close to the diffusion limit—which facilitates rapid assembly (40). However, experiments using hapten–antibody interactions have shown lower stability than biotin-streptavidin under force. For instance, a recent study of DNA tether lifetime using multiple digoxigenin–anti-digoxigenin interactions on a nitrocellulose coated surface measured a median value of ∼7 min at 45 pN (41). Covalent chemistries for DNA tethering have been developed but can suffer from slow reaction kinetics or elaborate and time-consuming surface modification requirements (41,42). Therefore, there remains a need for techniques which can enhance tether lifetime without the requirement for complicated experimental procedures.

**Figure 3.**
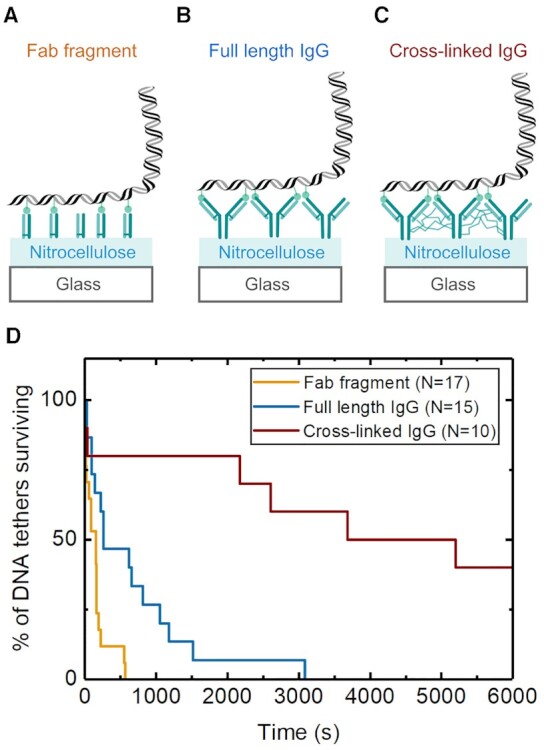
Tether lifetime enhancement by cross-linking of anti-digoxigenin antibodies. The lifetime of single DNA tethers at 40pN was measured for three different surface preparations: (**A**) Fab fragments, (**B**) full-length IgG antibodies and (**C**) full-length IgG antibodies cross-linked with glutaraldehyde. (**D**) Survival probability curves for the three surface preparation methods. The total number of single tethered DNA molecules measured is shown in the legend.

Tethering via anti-digoxigenin bound to a nitrocellulose surface relies on non-covalent bonds at two levels: (i) between the nitrocellulose and anti-digoxigenin antibody and (ii) between the binding sites of the antibody and digoxigenin labels on the DNA. Nitrocellulose binding to an antibody is widely used in western blotting and is thought to rely mainly on hydrophobic contacts with the protein (43). We reasoned that this interaction would likely be stabilized by increasing the surface area contact between the antibody and nitrocellulose. We therefore compared the tether stability for three different surface preparation methods: (i) anti-digoxigenin fab fragments, (ii) full-length anti-digoxigenin and (iii) full-length anti-digoxigenin combined with a glutaraldehyde crosslinking step (see Materials and Methods and Figure 3A–C). Glutaraldehyde reacts with both amine and cysteine residues creating a covalent link between neighbouring antibodies on the surface. After a short crosslinking step by incubation with glutaraldehyde, the flow cell was extensively washed with 1 M Tris (pH 7.5) to quench the crosslinking reaction.

We selected single DNA tethers by checking for the absence of DNA braiding upon rotation of the magnets (44). We found that the median tether lifetime at 40 pN increased from 158 s for fab fragments to 261 s for full-length antibodies which suggests that the larger surface area of the full-length antibody helps to stabilize binding (Figure 3D). We then found a substantial increase to a median value of 6780 s after adding the cross-linking step with glutaraldehyde further indicating stabilization of the interaction of the tether at the surface substrate. We did not observe a significant change in the proportion of torsionally-constrained tethers (measured with one DNA batch) which was 58% for the fab fragment surface, 52% for the full-length IgG surface and 50% for the full-length IgG surface with cross-linking. A wide range of DNA–protein interactions require force spectroscopy measurements at applied tension in range of tens of picoNewton. For instance, RNA polymerase has a stall force of ∼25 pN (45). DNA helicase measurements also typically use forces of 10–30 pN depending on the specific structure of the DNA template used (8). Therefore, this simple addition of a short crosslinking step should prove helpful for experiments seeking to collect large datasets at high forces.

### Hairpin construction with synthetic oligonucleotides

**Figure 4.**
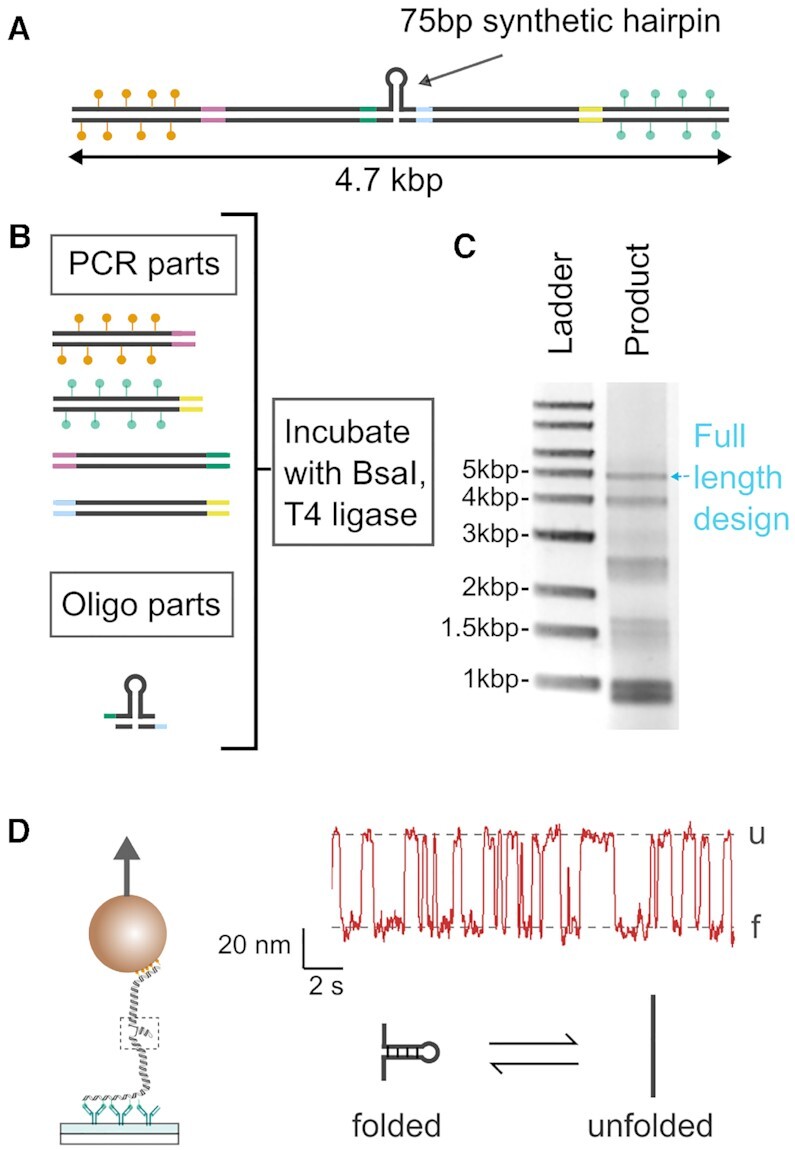
Construction of DNA with a short synthetic hairpin. (**A**) Design of construct with labelled biotin and digoxigenin handles and 75 bp synthetic hairpin at centre. (**B**) The design is assembled by separately preparing the PCR parts and oligo parts as described in Figure 1 before a one-pot incubation with BsaI and T4 DNA ligase. (**C**) Agarose gel image of product showing band corresponding to full-length construct. (**D**) Schematic of magnetic tweezers experiment and extension-time trace at 14 pN for one example bead. The extension shows spontaneous fluctuations between two states characteristic of the unfolding and refolding of the DNA hairpin.

Having demonstrated the efficient assembly of long, end-labelled dsDNA using golden gate assembly, we tested the possibility of constructing DNA structures which accommodate synthetic oligonucleotide parts. Specifically, we made a design which featured a short synthetic DNA hairpin positioned in the centre of the DNA. Such hairpin structures are particularly useful for studying the thermodynamics of DNA duplexes using magnetic and optical tweezers (46). Figure 4A shows a schematic of this design and Figure 4B shows the components used in the golden gate assembly. The assembled product was analysed by gel electrophoresis and showed a clearly defined band (Figure 4C) at the expected length of ∼5 kb. From image analysis of the gel, the amount of DNA in the final product band for this short hairpin construct is 7% of the total DNA added to the gel lane and the concentration of the product band after golden gate assembly was 6 ng/μl. Magnetic tweezers experiments showed the characteristic unfolding and refolding pattern of such a short DNA hairpin when a constant force of 14 pN was applied (47). The percentage of magnetic beads attached to the flow cell surface which showed this characteristic hairpin transitioning was 69% (Supplementary Table S9).

**Figure 5.**
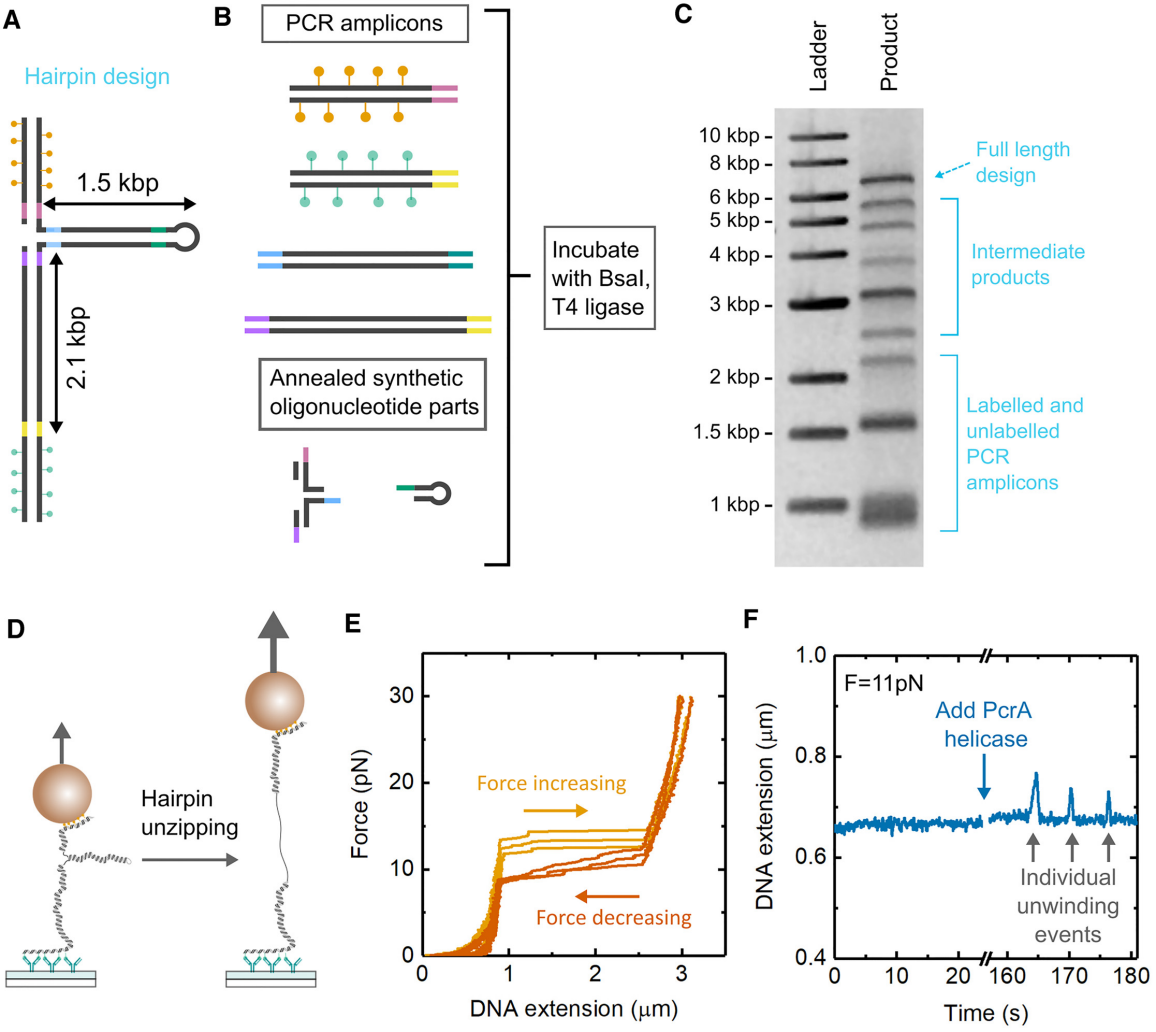
Large DNA hairpin construction. (**A**) Design of DNA hairpin with 1.5 kb duplex arm and 2.1 kb spacer between the bead and surface. (**B**) Four PCR amplicons are synthesized together with two oligonucleotide parts and incubated with BsaI and T4 DNA ligase. (**C**) Gel analysis of final product. Bands from the starting material PCR amplicons, together with intermediate products and the final full-length product are indicated. (**D**) Schematic of DNA hairpin assembled in a magnetic tweezers experiment. At high forces the hairpin section is unzipped. (**E**) Example traces from three separate beads showing force–extension curves during increasing and decreasing force ramps. (**F**) Single molecule unwinding events after addition of the helicase PcrA at a constant force of 11 pN.

We also synthesized a DNA construct which uses a larger 1.5 kb PCR amplicon as the duplex hairpin structure—Figure 5A and B. Longer DNA hairpins formed in this way have been widely used for studying helicase-mediated DNA duplex unwinding activity (48). The design incorporates four PCR amplicons and two oligonucleotide parts—a three way connector made by annealing four separate oligos and a single hairpin loop oligo. Figure 5C shows an agarose gel of the product after golden gate assembly. A full assignment of the bands in the gel is given in Supplementary Figure S4. The amount of DNA in the final product band is 10% of the total DNA added to the gel lane (as determined by gel analysis) and the concentration of the product band after golden gate assembly was 10 ng/μl.

Figure 5D shows a schematic of the hairpin design tethered in a magnetic tweezers experiment. High forces cause unzipping of the hairpin resulting in a large increase in DNA extension. Figure 5E shows example force-extension curves of three beads where this hairpin unzipping is clearly visible and forms a hysteresis loop as the force is ramped up and then down. The percentage of magnetic beads attached to the flow cell surface which showed such a hairpin transition was 65% (Supplementary Figure S5 and Supplementary Table S9). In Figure 5F, we show an example recording from one bead showing DNA extension as a function of time after adding PcrA helicase to the flow-cell. PcrA (in the absence of its helper protein RepD) is a weakly processive, type 1A superfamily 3′-5′ DNA helicase found in gram positive bacteria which has roles in DNA repair and plasmid replication (49,50). The connector section features a 12 thymine overhang for loading of PcrA so that it can bind and subsequently unwind the duplex hairpin. After addition of the PcrA, saw-tooth changes in DNA extension are observed indicating PcrA unwinding creating DNA extensions on the order of 50 nm followed by rapid reannealing after PcrA dissociates.

## DISCUSSION

The recent drive to assemble long double-stranded DNA *in vitro* for genome assembly has led to the development of several techniques such as Gibson assembly and golden gate assembly (51,52). Gibson assembly uses the concerted actions of a DNA exonuclease, DNA polymerase and DNA ligase whereas golden gate assembly uses a Type IIs restriction enzyme combined with DNA ligase (53). The lack of an exonuclease/polymerase step means that golden gate assembly is able to accommodate synthetic oligonucleotides at designed positions during a one-pot test-tube assembly. As we describe in this paper, golden gate assembly is therefore ideally suited to the fabrication of DNA constructs requiring synthetic oligonucleotide motifs such as the DNA hairpins demonstrated here and could be easily extended to incorporate modifications such as fluorophores or modified bases at designed positions. Furthermore, we have shown that high yields of long (10 kbp) DNA can be achieved by combining multiple shorter PCR fragments—suitable for fluorescence studies on flow-stretched DNA and investigation of torsional properties using magnetic tweezers. A slight disadvantage of our method is that the use of PCR fragments may introduce a small number of errors in sequence fidelity and therefore for some techniques where purely dsDNA constructs with end labels are needed, the widely used technique of plasmid amplification and ligation may be more appropriate (54–57). Another consideration is that the DNA amplified by PCR should not contain internal restriction sites for the specific Type IIs enzyme used and also the four base sequences of the overhangs are unavoidably included in the final sequence of the structure.

Golden gate assembly has proven useful for large scale assemblies of DNA plasmids and genomes. By improving the efficiency of Type IIs restriction enzymes and T4 DNA ligase activity, several recent advances have been made including the generation of genomes from up to 52 parts by combining *in vitro* assembly and genetic selection (33). We anticipate that further optimization of the methods shown here will be useful for generating longer DNA, up to 10 s of kbps, that can be of used in fluorescence studies where very long DNA substrates are needed. Overall, the methods demonstrated here greatly simplify the preparation of DNA substrates for single molecule biophysical studies.

## DATA AVAILABILITY

All relevant data is available in the paper or the Supplementary Information.

## Supplementary Material

gkac300_Supplemental_File
