# Supplementary material for: Efficient golden gate assembly of DNA constructs for single molecule force spectroscopy and imaging
Source: Nucleic Acids Res. 2022 Apr 30;50(13):e77. doi: 10.1093/nar/gkac300 (PMC9303394; doi:10.1093/nar/gkac300)
Supplement: gkac300_Supplemental_File [file gkac300_supplemental_file.pdf]

SUPPLEMENTARY INFORMATION

Efficient golden gate assembly of DNA constructs for single molecule force spectroscopy and imaging

Nicholas A. W. Bell[1,*], Justin E. Molloy[1,*]

[1] The Francis Crick Institute, London, NW1 1AT, United Kingdom

*To whom correspondence should be addressed. Tel: +44 (0) 203 796 2352; Email: nicholas.bell@crick.ac.uk, justin.molloy@crick.ac.uk

Tables:

Part	Forward primer	Reverse primer
Biotin handle (860 bp)	GAT**GGTCTC**ATGCCGCAACAGCACAACCCAAACTG	AATCTGCTGCAATGCCACAG
2.1 kbp section 1	GAT**GGTCTC**AGGCAGCCACTTCAGCACGAGATG	GAT**GGTCTC**AAAGGTCCAGATCTGCAGCATCCTGAA
2.1 kbp section 2	GAT**GGTCTC**ACCTTGCCACTTCAGCACGAGATG	GAT**GGTCTC**ATAGATCCAGATCTGCAGCATCCTGAA
2.1 kbp section 3	GAT**GGTCTC**ATCTAGCCACTTCAGCACGAGATG	GAT**GGTCTC**ACTACTCCAGATCTGCAGCATCCTGAA
2.1 kbp section 4	GAT**GGTCTC**AGTAGGCCACTTCAGCACGAGATG	GAT**GGTCTC**AGCTCTCCAGATCTGCAGCATCCTGAA
Digox. handle (860 bp)	GAT**GGTCTC**AGAGCGCAACAGCACAACCCAAACTG	AATCTGCTGCAATGCCACAG

Table S1. Primer sequences for PCR parts used in 10.1 kbp end-labelled duplex design. The PCR parts of Figure 2A were amplified using λ-DNA as the template. The primer sequences are shown in Table S1. The BsaI recognition site is highlighted in bold. The four base overhang generated by BsaI digestion is shown in colour. The four base sequences in the designs were chosen based on recommended sets from the NEB golden gate assembly tool (https://goldengate.neb.com/). Complementary sequences share the same colour.

Part	Part stoichiometry
Biotin handle (860 bp)	8
2.1 kbp section 1	1.2
2.1 kbp section 2	1
2.1 kbp section 3	1
2.1 kbp section 4	1.2
Digoxigenin handle (860 bp)	8

Table S2. Stoichiometry for golden gate reaction used in 10.1 kbp design. In the golden gate reaction shown in Figure 2 in the main text, the six parts were mixed in the above stoichiometric ratios

---

Part	Forward primer	Reverse primer
Biotin handle (860 bp)	GAT**GGTCTC**ATGCCGCAACAGCACAACCCAAACTG	AATCTGCTGCAATGCCACAG
1.5 kbp section 1	GAT**GGTCTC**AGGCACCGTGGAATGAACAATGGAAGTC	GAT**GGTCTC**AACCTCTGTCAGGTGGCTCAATCTCTTC
1.5 kbp section 2	GAT**GGTCTC**AAGAGGCCACTTCAGCACGAGATG	GAT**GGTCTC**AGCTCTCCAGATCTGCAGCATCCTGAA
Digox. handle (860 bp)	GAT**GGTCTC**AGAGCGCAACAGCACAACCCAAACTG	AATCTGCTGCAATGCCACAG

Table S3. Primer sequences for PCR parts used in short hairpin design. The DNA hairpin structure of Figure 4 is composed of four PCR parts and one oligonucleotide part. The primers for the PCR parts (using λ-DNA as the template) are shown.

Oligo	Sequence
Oligo1 – short hairpin	PhosAGGTTTCGCTGGGATCCAAGCGAAGTGTCTACTATCCCTAAGCCCATTTCTCGCACAATAACCCCTGAATG TGTCCGCATCTGATGTTACCCGGGTTTTTACCCGGGTAACATCAGATGCGGACACATTCAGGGGTTATTGTGCG AGAAATGGGCTTAGGGATAGTAGACACTTCGCGTCTGTGACTAACTG
Oligo2 – short hairpin	GCTTGGATCCCAGCGAA
Oligo3 – short hairpin	PhosCTCTCAGTTAGTCACAGACGC

Table S4. Oligonucleotide sequences used for oligonucleotide part in short hairpin design. The hairpin is assembled by annealing the three oligonucelotides: Note: Oligo1 was a DNA ultramer from IDT. “Phos” indicates a 5’ phosphorylation.

Part	Part stoichiometry
Biotin handle (860 bp)	2.25
1.5 kbp section 1	1.5
Oligo part	1
1.5 kbp section 2	1.5
Digoxigenin handle (860 bp)	2.25

Table S5. Stoichiometry for golden gate reaction used in short hairpin design. In the golden gate reaction, the five parts were mixed in the corresponding stoichiometric ratios.

Part	Forward primer	Reverse primer
Biotin handle (860 bp)	GATGGTCTCATGCCGCAACAGCACAACCCAAACTG	AATCTGCTGCAATGCCACAG
1.5 kbp section	GATGGTCTCAGCAACCGTGGAATGAACAATGGAAGTC	GATGGTCTCATCTGCTGTCAGGTGGCTCAATCTCTTC
2.1 kbp section	GATGGTCTCAAGGAGCCACTTCAGCACGAGATG	GATGGTCTCAGCTCTCCAGATCTGCAGCATCCTGAA
Digox. handle (860 bp)	GATGGTCTCAGAGCGCAACAGCACAACCCAAACTG	AATCTGCTGCAATGCCACAG

Table S6. Primer sequences for PCR parts used in long hairpin design. The DNA hairpin structure of Figure 5 is composed of four PCR parts and two oligonucleotide parts. The primers for the PCR parts (using λ-DNA as the template) are shown.

Oligo	Sequence
Oligo1 - connector	PhosGGCATTCGCTGGGATCCAAGCTTGGCAGCTGAAACCGCC
Oligo2 - connector	GCTTGGATCCCAGCGAA
Oligo3 - connector	PhosTTGCGGCGGTTTCAGCTGCCATTTTTTTTTTTTTCGTCTGTGACTAACTG
Oligo4 - connector	PhosTCCTCAGTTAGTCACAGACGA
hairpin loop	PhosCAGATTGCCAAGTGAGTCCGATTTTTCGGACTCACTTGGCAA

Table S7. Primer sequences for PCR parts used in long hairpin design. "Phos" indicates a 5' phosphorylation. The three-way connector oligonucleotide is assembled by annealing the four oligonucelotides while the hairpin loop sequence is separately annealed by itself.

Part	Part stoichiometry
Biotin handle (860 bp)	2.4
1.5 kbp section	1.2
Oligo connector part (4 annealed oligos)	1
2.1 kbp section	1.2
Oligo hairpin part (1 annealed oligo)	5
Digoxigenin handle (860 bp)	2.4

Table S8. Stoichiometry for golden gate reaction used in long hairpin design. In the golden gate reaction, the five parts were mixed in the above stoichiometric ratios.

Design	Total single beads	# good tethers	% good tethers
10 kbp dsDNA (batch 1)	29	15	52
10 kbp dsDNA (batch 2)	86	35	41
10 kbp dsDNA (batch 3)	65	18	28
Short hairpin	201	139	69
Long hairpin	213	139	65

Table S9. Summary of statistics on DNA tethers. Three separate batches of the 10 kbp dsDNA design were used (Figure 2). For this design a "good" tether is one which is torsionally constrained. For the hairpin designs, a "good" tether is one for which there is the expected signal due to unfolding of the hairpin.

FIGURES:

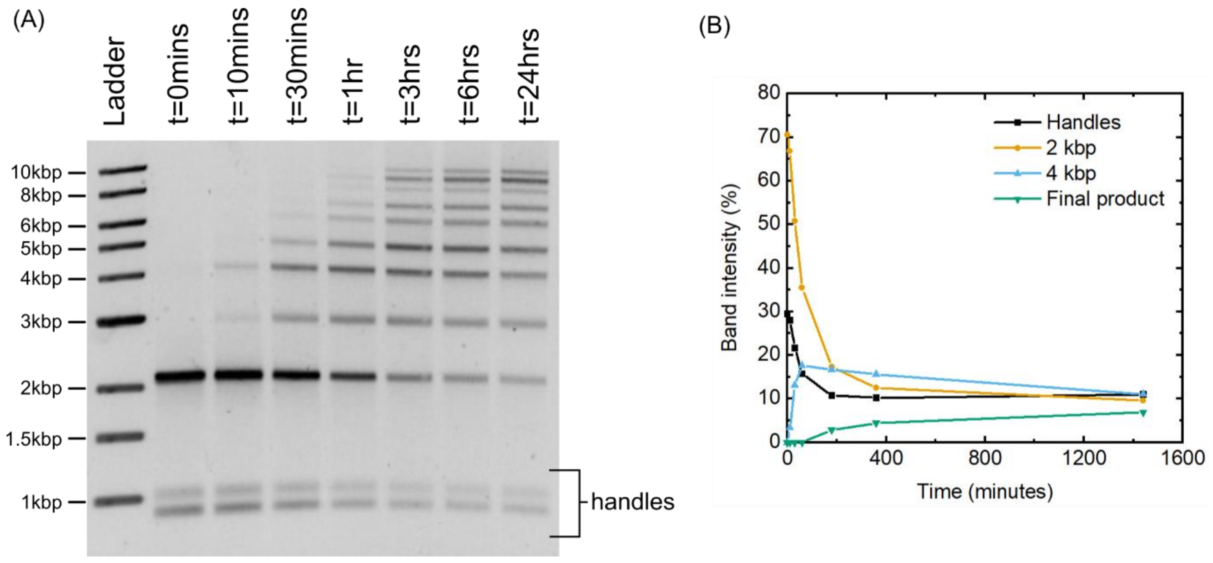

Figure S1. Time dependence of golden gate DNA assembly. The DNA was mixed as in Table S2 (except a stoichiometric ratio of 2.4 was used for the handles). (A) The time indicates the length of assembly incubation. The reactions were quenched at each timepoint with 1x SDS loading dye (NEB). (B) Quantitation of band intensity (as % of total signal from all bands at a given timepoint). The % of the final product (10 kbp) at t= 24 hrs is 6%.

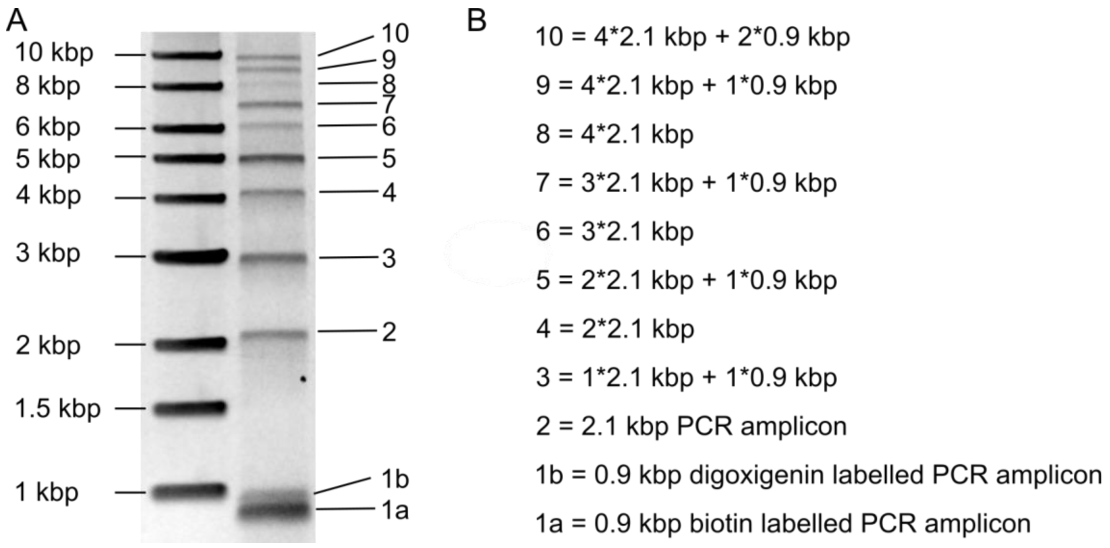

Figure S2. Full assignment of gel bands from 10.1 kbp DNA product. Gel is as shown in Figure 2B in main text. (A) The ladder markers are labelled on the left and the bands in the product numbered on the right. (B) Assignment of different bands from starting materials (1a, 1b and 2) to full length (10).

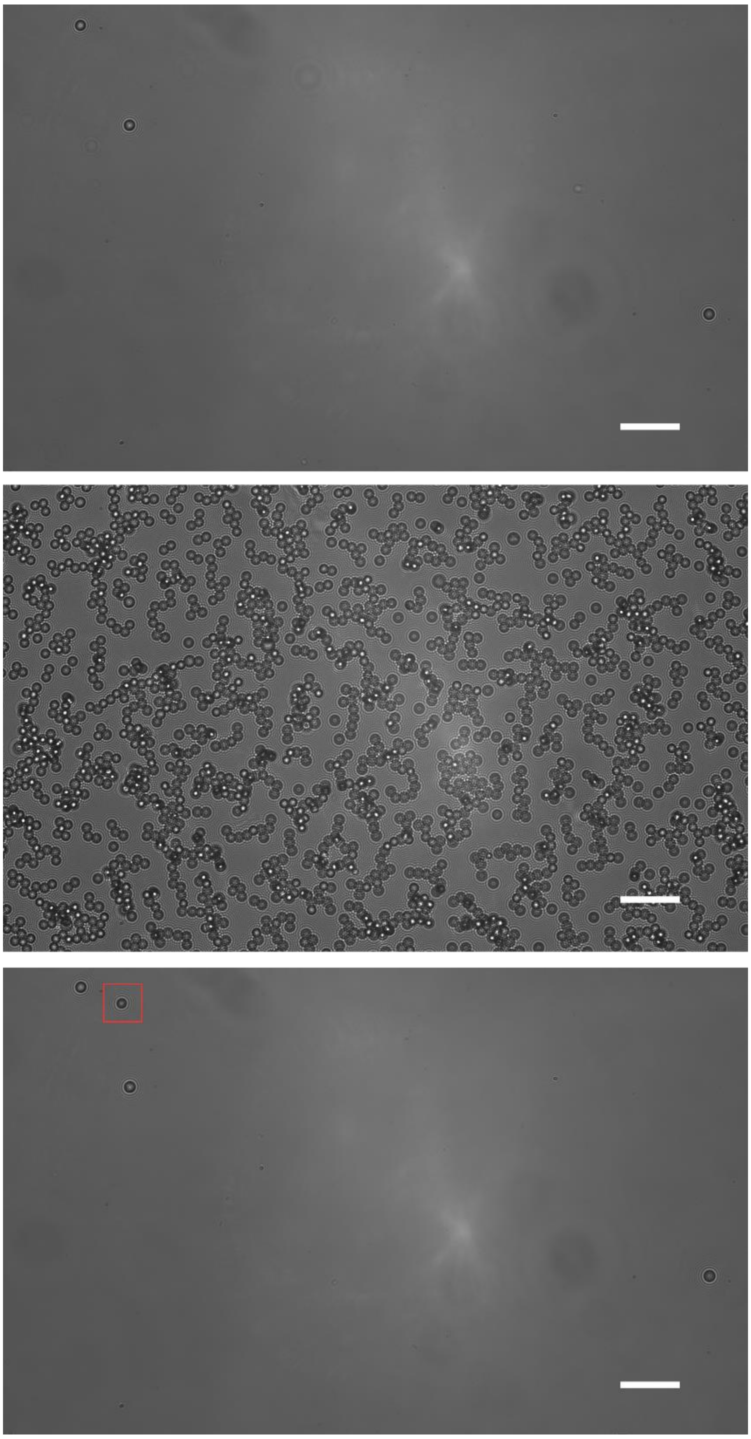

Figure S3. Test of non-specific attachment on flow-cell surface. A flow-cell surface was prepared as described in the Materials and Methods using full length anti-digoxigenin and a glutaraldehyde fixing step. The top image shows three fiducial beads in the field of view which are used for drift correction. For the test, 10 µL Dynabead M280 stock was mixed with 10 µL 2 mg/ml BSA, 2 mg/ml β-casein in 20 mM Tris-HCl (pH 7.5), 150 mM NaCl and 5 µL of this mixture was added into the flow-cell (middle image). This mimics typical conditions used with DNA coated beads. After incubating for five minutes, ~20 channel volumes of 1 mg/ml BSA, 1 mg/ml β-casein in 20 mM Tris-HCl (pH 7.5), 150 mM NaCl was passed through the channel. The bottom image shows the same field view after washing – there was one non-specifically attached magnetic bead which is marked in red. From five such fields of view we measured a total of three non-specifically attached beads. The scale bar is 20 µm.

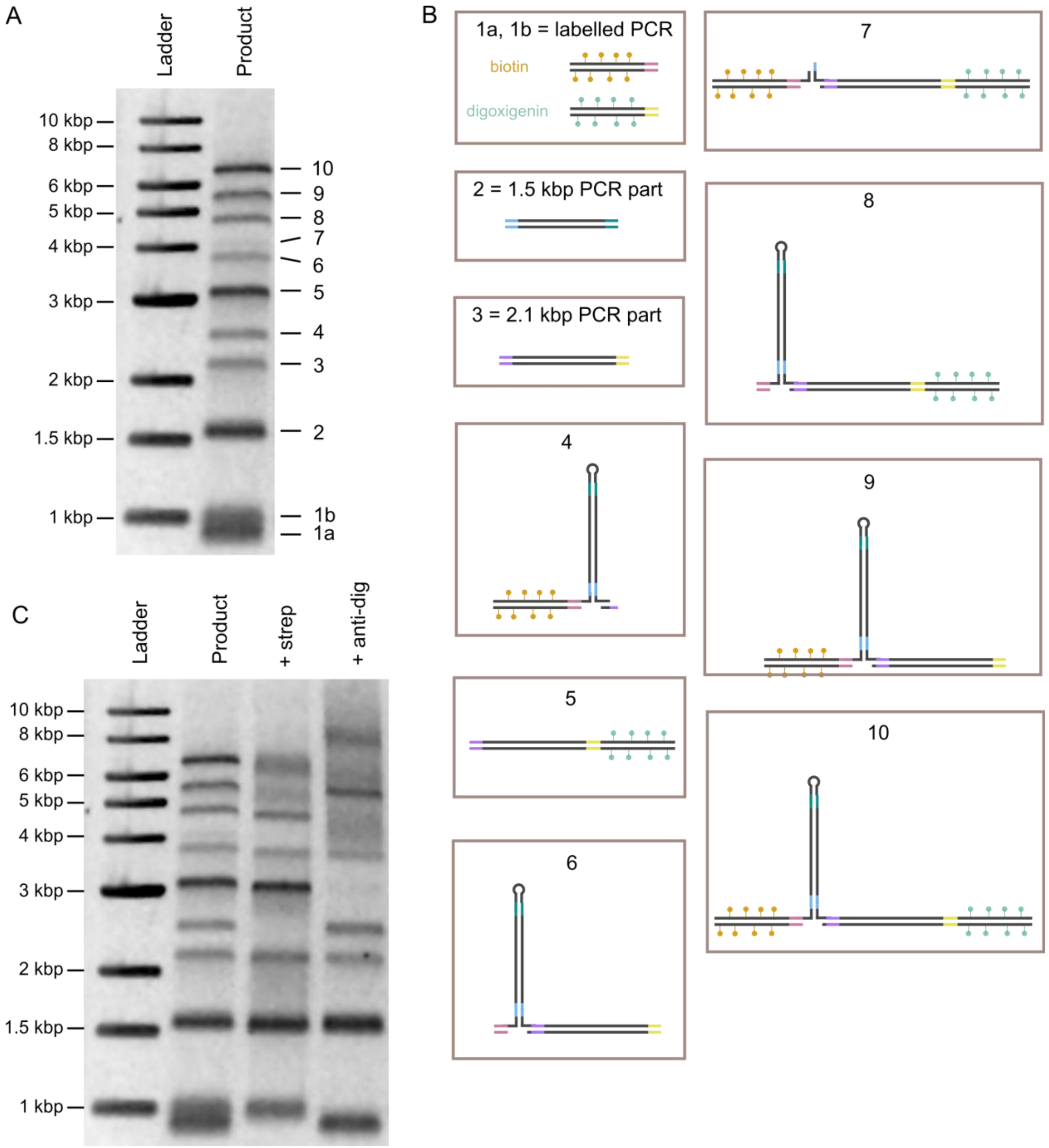

Figure S4. Full assignment of gel bands from long DNA hairpin. Gel is as shown in Figure 5C in main text. (A) The bands in the product are numbered on the right and the ladder markers are indicated on the left. (B) Assignment of different bands in product. The small oligonucleotide parts may or may not be present at the end since their small size means their presence/absence would not cause an appreciable band shift. (C) Same gel as in (A) but subsequent lanes are also shown where either streptavidin or anti-digoxigenin has been added to help to identify the bands.

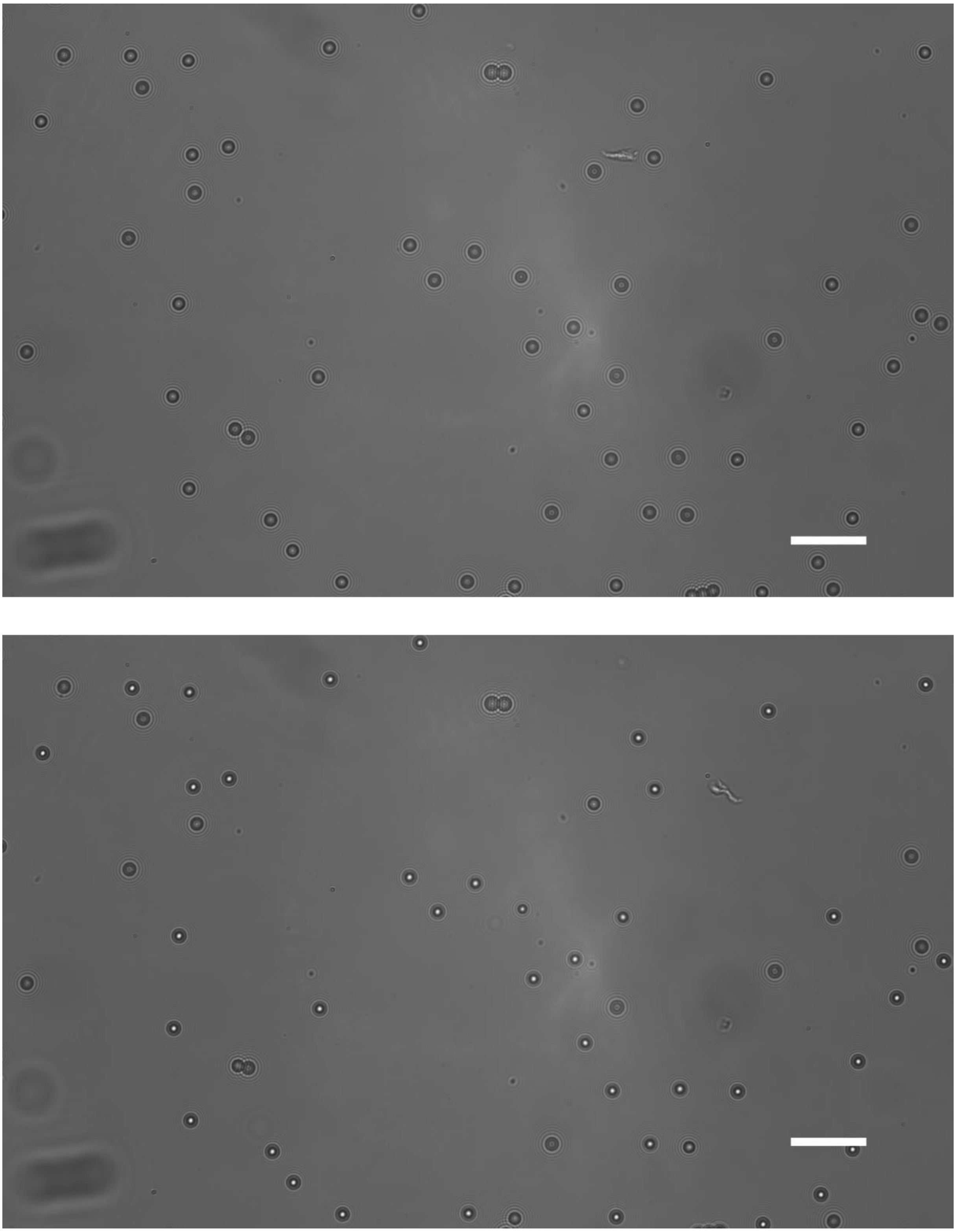

Figure S5. Visualization of unfolding of the large DNA hairpin construct. The images show a typical field of view at 0.01 pN (top image) and 30 pN (bottom image) (see also Figure 5 main text) At the objective focus used here, the beads undergoing a hairpin unfolding transition are observed as having a whiter centre due to the change in diffraction pattern. The scale bar is 20 µm.

Lab protocols for PCR synthesis, oligo annealing and golden gate assembly

(A) **Handle synthesis:** The labelled PCR amplicons (DNA “handles”) for surface attachment were made by mixing the following (using the NEB Taq PCR kit - E5000S):

10 µL 10x standard taq buffer
2 µL dNTP mix @ 10 mM
0.5 µL lambda DNA @ 5 ng/µL
2.5 µL forward primer @ 10 µM
2.5 µL reverse primer @ 10 µM
2.5 µL modified dUTP (digoxigenin or biotin) @ 1 mM
79.5 µL ddH_2O
0.5 µL Taq polymerase @ 5 U/µL

The PCR protocol was as follows:

1) 95 ºC for 60 s
2) 95 ºC for 30 s
3) 54 ºC for 30 s
4) 68 ºC for 90 s
5) Repeat steps 2-4 x34
6) 68 ºC for 300 s

The product was purified with a Qiagen Qiaquick spin purification column, according to the manufacturers’ instructions, with typical yields of 100-200 ng/µL.

(B) **Unlabelled PCR amplicon synthesis:** PCR amplicons without labels were made using the NEB LongAmp Hot Start Taq2x master mix - M0533L with the following amounts:

25 µL 2x master mix
1 µL forward primer @ 10 µM
1 µL reverse primer @ 10 µM
1 µL lambda DNA @ 5 ng/µL
22 µL ddH_2O

The PCR protocol was as follows:

1) 94 ºC for 30 s.
2) 95 ºC for 20 s.
3) 58 ºC for 40 s.
4) 65 ºC for 50 s per kb.
5) Repeat steps 2-4 x30
6) 65 ºC for 600 s.

The product was purified with a Qiagen Qiaquick spin purification column, according to the manufacturers’ instructions, with typical yields of 100-200 ng/µL.

(C) **Oligo annealing**: Oligos were purchased from Integrated DNA Technologies and resuspended in IDT DNA duplex buffer (30 mM HEPES, pH 7.5; 100 mM potassium acetate). A particular oligo part was assembled by mixing all oligos together at equal stoichiometry with IDT DNA duplex buffer such that the final concentration of each oligo was 10 µM and then heating to 70 °C for one minute before linearly cooling to room temperature over 30 min.

(D) **Golden gate assembly**: The purified PCR amplicons and annealed oligo parts were mixed together at the ratios given above (Tables S2, S5, S8) together with BsaI-HFv2 (NEB - R3733S), T4 DNA ligase (NEB - M0202S) in 1x T4 DNA ligase buffer (NEB - B0202S). The assembly was carried out in a single tube of 20-40 µL volume with final concentrations of 1 U/µL BsaI-HFv2 and 20 U/µL T4 DNA ligase. The total DNA concentration in the golden gate reaction was 30-100 ng/µL and was kept as high as possible to maximise the ligation efficiency. The mixture was incubated for 3 hrs cycling between 37 °C and 16 °C every 5 min.
